# *Toxoplasma gondii* Infection Is Associated with Low Birth Weight: Findings from an Observational Study among Rural Bangladeshi Women

**DOI:** 10.3390/pathogens11030336

**Published:** 2022-03-10

**Authors:** Irin Parvin, Sumon Kumar Das, Shahnawaz Ahmed, Aminur Rahman, Abu Sadat Mohammad Sayeem Bin Shahid, Lubaba Shahrin, Farzana Afroze, Mst. Mahmuda Ackhter, Tahmina Alam, Yasmin Jahan, Parag Palit, Mohammad Habibur Rahman Sarker, Jui Das, Mohammad Enamul Hoque, Ricardo J. Soares Magalhães, Abdullah Al Mamun, Abu Syed Golam Faruque, Tahmeed Ahmed, Mohammod Jobayer Chisti

**Affiliations:** 1 International Centre for Diarrhoeal Disease Research, Bangladesh (icddr,b), Dhaka 1212, Bangladesh; irin.parvin@icddrb.org (I.P.); draminur@icddrb.org (A.R.); sayeem@icddrb.org (A.S.M.S.B.S.); lubabashahrin@icddrb.org (L.S.); farzanaafroz@icddrb.org (F.A.); mahmuda.ackhter@icddrb.org (M.M.A.); drtahmina@icddrb.org (T.A.); dr.yasminjahan@gmail.com (Y.J.); parag.palit@icddrb.org (P.P.); habibur.rahman@icddrb.org (M.H.R.S.); tahmeed@icddrb.org (T.A.); chisti@icddrb.org (M.J.C.); 2 Menzies—School of Health Research, Charles Darwin University, Darwin, NT 0811, Australia; 3 School of Health and Rehabilitation Sciences, The University of Queensland, Brisbane, QLD 4067, Australia; shahnawaz.ahmed@uq.net.au; 4 Mater Research Institute, The University of Queensland, Brisbane, QLD 4067, Australia; jui.das@uq.net.au; 5 NHMRC Clinical Trials Centre, The University of Sydney, Sydney, NSW 2006, Australia; mohammad.hoque@uq.net.au; 6 Institute for Social Science Research, The University of Queensland, Brisbane, QLD 4067, Australia; mamun@sph.uq.edu.au; 7 UQ Spatial Epidemiology Laboratory, School of Veterinary Science, The University of Queensland, Gatton, QLD 4343, Australia; r.magalhaes@uq.edu.au; 8 Children’s Health and Environment Program, UQ Child Health Research Centre, The University of Queensland, Brisbane, QLD 4067, Australia

**Keywords:** Bangladesh, low birth weight, pregnant women, rural, *Toxoplasma gondii*, *T*. *gondii*

## Abstract

Gestational *Toxoplasma gondii* (*T. gondii*) infection may cause substantial adverse effects on developing fetuses, newborns and also mothers. This study aims to estimate the seroprevalence of *T. gondii* among rural Bangladeshi pregnant women and determine the risk of a low birth weight (LBW). We followed a longitudinal design where 208 pregnant women were followed until the birth of their infants. Levels of IgG and IgM of *T. gondii* were assessed using chemiluminescent immunoassay. Modified Poisson regression was used to estimate crude and adjusted associations and multiple regression analysis was performed to understand the confounding and modifying effects of the variables. Thirty-nine (19%) children were born with LBW, among whom 15 (39%) mothers were positive for *T. gondii* IgG during pregnancy. After adjusting for several confounders and modifiers, pregnant women with *T. gondii IgG* or IgM seropositivity were significantly associated with LBW of infants (aRR: 2.00, 95% CI: 1.17–3.42). The strength of this association increased after adjusting for maternal education (aRR: 4.88, 95% CI: 1.74–13.69). The final model had an AROC of 0.84 with a sensitivity of 36% and specificity of 97%. Although causality is yet to be established, the study observed an association between *T. gondii* infection during pregnancy among rural Bangladeshi women and LBW of newborns.

## 1. Introduction

*Toxoplasma gondii* (*T. gondii*) is well known to be a globally endemic zoonotic parasitic disease which is transmitted through contaminated food or water or undercooked meat [1,2]. Infection during pregnancy may lead to several fetal complications, including fatal and adverse outcomes of conception, such as abortion, stillbirth, intrauterine death, preterm delivery or congenital toxoplasmosis [3]. However, a causal relationship is heterogenic and inconclusive, since many other concurrent factors contribute in a different way to the development of these adverse and fatal outcomes [4,5], including low birth weight (LBW) among newborns [6,7,8,9,10]. Untreated toxoplasmosis may lead to serious clinical manifestations during childhood or early adulthood [11].

A recently published meta-analysis reported that vertical transmission of *T. gondii* is not unusual, and the rate has been reported to increase up to 32% during the 3rd trimester [12]. However, these studies failed to determine a potential relationship between *T. gondii* infection and fetal outcomes and LBW of newborn babies [13,14]. Hence, the potential risk of adverse outcomes to the fetus, including LBW, is divisive and unclear due to a gross lack of epidemiological data [7,10]. Henceforth, further research for reliable estimates of maternal *T. gondii* seroprevalence is warranted.

Most importantly, *T. gondii* infection remains undiagnosed because of its asymptomatic nature, lack of resources, and least priority in the health care policy, especially routine screening or surveillance [11,15]. Globally, a wide variation in the prevalence of *T. gondii* infection has been reported ranging from 10% to 80% [16]. Among the low- and middle-income countries, a few studies were conducted in Bangladesh with notable variation in the *T. gondii* infection rate (*ranged from 35% to 55%*) [17,18].

Although Bangladesh has made good progress in reducing child mortality and improving maternal and child nutrition, the prevalence of LBW in newborns remains high (23%) compared to the global prevalence of 15% [19,20]. It is now widely reported as the immediate, short-term and long-term health and developmental risk for children born with LBW [21,22,23]. Due to the high prevalence of LBW newborns in Bangladesh [24], the identification of high-risk pregnancies might have a greater advantage for essential screening. Moreover, the LBW is multifactorial and many of these factors share the risk of *T. gondii* infection.

Due to the high prevalence of LBW among newborns in Bangladesh [24], the identification of high-risk pregnancies may impart a greater advantage for an essential screening program to combat the challenge of routine sero-screening of *T. gondii.* Given the paucity of reports on these issues, the current study aimed to estimate the seroprevalence of *T. gondii* among rural Bangladeshi pregnant women and to ascertain their risk of giving birth to newborns with LBW.

## 2. Results

Nineteen percent of the newborns were identified as LBW newborns, irrespective of their gestational age. Thirty eight point five percent of the pregnant women who delivered LBW babies were positive for *T. gondii* IgG compared to 17.8% of pregnant women positive for *T. gondii* IgG but delivered a baby of normal birth weight (Table 1). None of the pregnant women who delivered LBW babies were positive for both *T. gondii* IgG and IgM or only positive IgM. 

Table 2 demonstrates the detailed distribution of different maternal characteristics and their crude association with LBW newborns. Maternal education of less than primary schooling was found to be associated with LBW. For other co-variates, mild-to-moderate associations were observed, although none of these associations were significantly different.

Pregnant women sero-positive for *T. gondii* IgG or IgM were significantly associated with LBW (Table 3). The minor modifying effect was observed after adjusting for several maternal sociodemographic profiles (Model 2), pregnancy characteristics (Model 3) and pregnancy BMI category (Model 4). In the final model (Model 5), after adjusting for gestational age at enrolment and term pregnancy, the association between *T. gondii* IgG or IgM seropositivity and LBW among newborns was found to increase, and it was significantly different (aRR-2.00; this model of explained 19.5% variance would be the best fit model due to the lowest Akaike’s information criteria (AIC) and relatively higher Pearson’s goodness-of-fit (*p* = 0.948)). In the final adjusted model, a significant association was found between the birth of LBW babies and less than primary schooling of mothers and the middle wealth index. As a consequence, maternal education was found as the only potential modifier, and adjusting for interaction with *T. gondii* IgG or IgM sero-positive, the association increased (aRR: 4.88 (95% CI: 1.74–13.69)) and remained significant with a relatively wider confidence interval (Supplementary Table S1). After excluding IgM+; IgG+ and IgM+; IgG−, the adjusted model (Model-5), also showed a significant association (aRR: 2.46 (1.42–4.25)) (Supplementary Table S2). For other pathogens of the TORCH panel (rubella, cytomegalovirus, herpes simplex-1 and 2), there was no significant association with LBW (details under Supplementary Table S3).

**Figure 1:**
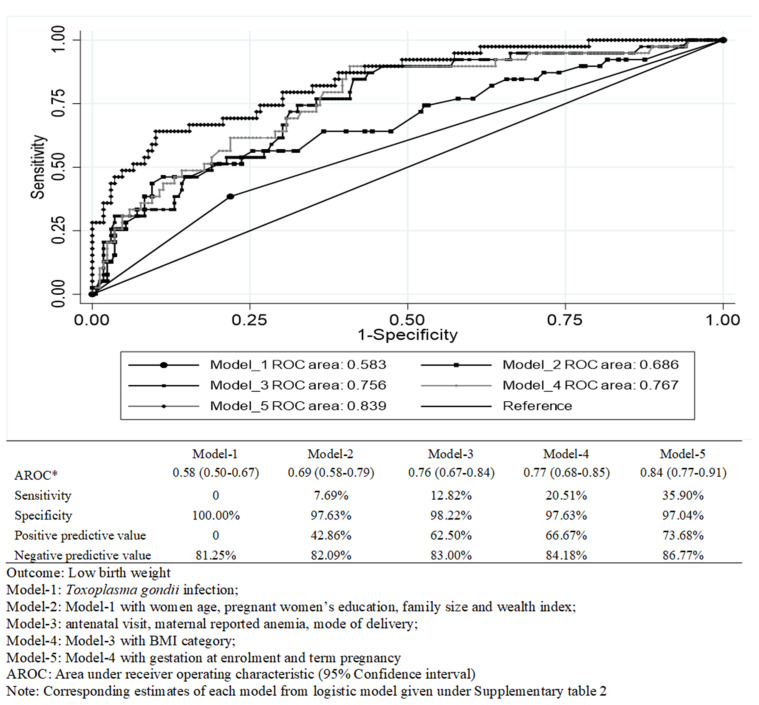
Discriminatory capacity of the models.

Model 1 in Figure 1 showed very low discriminatory capacity (0.58) of *T. gondii* infection to predict LBW with 0% sensitivity, indicating no clinical importance. However, Model-5 in Figure 1 showed a high discriminatory capacity of 0.84 with a sensitivity of 36%; thus, indicating that if a mother had these specific characteristics, screening for *T. gondii* may be worthwhile for the early prediction of LBW. (Corresponding goodness-of-fit estimated from logistic regression models incorporated under Supplementary Table S4)

## 3. Discussion

Based on the current knowledge and existing evidence, the true association between *T. gondii* infection during pregnancy with LBW is still unclear [6,7,8,9,10]. One of the major limitations that these studies have acknowledged is the lack of good data following robust study designs, including robust assessment of other confounding and modifying factors related to *T. gondii* infection and LBW. The other challenge is the gross variation in the prevalence of *T. gondii* infection during pregnancy due to geo-climate diversity, environmental distinctiveness and maternal behaviors, such as hygiene practices before and during pregnancy [25]. Nevertheless, the current study’s findings opened the window of further research by establishing a relatively strong association between *T. gondii* infection and LBW newborns in a resource-poor country like Bangladesh, where accessing the fundamental antenatal care is still a concern, especially in a rural, remote area [26,27].

Although it is yet to be fully understood—or that the association does not establish the causality—the relative risk remains unchanged, even after adjusting for many other contributing factors for LBW. Moreover, after adjusting for term pregnancy and gestation at enrollment (point of *T. gondii* serological test, meaning that serum was collected just after enrollment), the relative risk between *T. gondii* infection and LBW increased from 1.78 to 2.00. The explanation of such a relatively strong association was underpinned due to a lack of additional data, such as an active *T. gondii* infection related to congenital toxoplasmosis, which is associated with a greater risk of congenital anomalies [28]. However, the prevalence of reported congenital toxoplasmosis was very low (0.3/1000 live birth) globally [29] and none of our study children were identified to be having any congenital anomaly.

Overall, the prevalence of *T. gondii* infection in our study population was 25% (52/208); however, the prevalence of *T. gondii* IgM−; IgG+ was relatively higher (38%, 15/39) among enrolled mothers who delivered newborns with LBW. Such overall high prevalence might be closely similar to some other Asian countries, such as Yemen (21.2%) [17], Saudi Arabia (24.1%) [30] and Myanmar (31.7%) [31]. However, it does not establish that the primary infection during pregnancy is unlikely because of previous *T. gondii* exposure. Moreover, in many other countries where *T. gondii* seroprevalence remains very high, such as 85.3% in Ethiopia, 67.0% in Jordan, 58.3% in Turkey and 42.5% in Malaysia, there is a gross lack of data on the impact of *T. gondii* infection on the fetus and newborn [32,33,34,35]. The major gap is limited, and there is inconclusive evidence on the potential benefits to women and babies of routine testing for *T. gondii* infection, and it is not cost-effective even in developed countries [36].

There is still no clear pathological evidence to understanding the vertical transmission of *T. gondii* infection and the possible mechanism for LBW in newborn babies. Eating raw or insufficiently cooked meat, improper hygiene practices, such as handwashing after handling raw meat, vegetables or fruits, and contact with domestic pets are the major paths of *T. gondii* transmission [37,38]. Conversely, the risk of LBW is multifactorial, including maternal host and behavioral factors, several sociodemographic factors, and certain environmental exposures [39]. The current study made an attempt to accumulate the available various types of factors related to LBW, especially age, economic backgrounds, such as the wealth index and nutritional status. Uncertainty remains in confounding or mediating effects on the overall association between *T. gondii* infection and LBW. However, the systematic association confirmed no potential confounding and modifying effect, except for pregnant women’s education. Women’s education is one of the fundamental and common predictors for several maternal and child outcomes [40]. The modifying effect of maternal education might be correlated with other factors, such as hygiene knowledge and practice, healthy food consumption, care during pregnancy, including antenatal care, and we have adjusted some of them in our current study.

It remains tough to predict the potential impact of the current research findings in set-ups of resource-poor Bangladesh, because of existing antenatal healthcare policy and practice, the cost of screening and the asymptomatic nature of infections. Although a single rural set-up with a small sample size does not represent the country’s population, to our knowledge, this was the first study aimed at determining the current secondary objective. The prevalence of LBW is relatively lower compared to the other studies in Bangladesh [39] which might be explained by the ongoing demographic surveillance system and the study population being supported by a very well known tertiary level hospital with well-established ante-natal and maternity care.

The study was conducted in a rural area where the contamination of sources is highly predominant due to the free movements of household pets and livestock. The widespread consumption of raw vegetables in rural areas due to the increasing production of crops and harvesting enhances the vulnerability to *T. gondii* infection among pregnant women. However, we do not have any environmental data on the rate of presence of oocysts in pet feces, especially cats, or the risk of contamination with water and food, and the presence of cysts in raw meats. Thus, the potential threat remains undiagnosed in the rural population and needs further zoonotic and environmental studies. 

### Limitations and Strengths

The current study had other limitations. Due to the lack of a favorable antibody profile, we were unable to determine the IgG avidity index in this study, which was considered the main differentiating indicator of acute phase infection from chronic phase infection. The analysis of secondary data was often unable to include many other co-variates associated with babies born with LBW, such as maternal pre-pregnancy characteristics [41], pregnancy behaviors [42,43], and pre-existing clinical or health conditions [44,45,46]. Many well-established factors were not significantly associated with LBW, such as maternal undernutrition, teenage motherhood, poor antenatal visits, anemia, and worsening wealth quintiles, which might be due to a lack of adequate power of the study, as reflected by the small sample size. Measurement of maternal pregnancy weight and height, instead of pre-pregnancy measurement, would be another limitation of the study. The estimated association between *T. gondii* infection and LBW in newborns might suffer from a statistical model overfitting (especially Model-5 under Table 3 and the interaction model under Table S1). It was challenging to test any such possibility due to inadequate sample size, to perform internal validity of the association between *T. gondii* infection and LBW in newborns across test and training data. However, to overcome these issues, to some extent, goodness-of-fit tests, including discriminatory capacity (AROC), were performed (see the details under Table 3, Tables S1 and S4). There was a clear indication that among the explanatory variables, preterm pregnancy had maximum weight in the final Model-5 (Table 3), which is biologically plausible, although we considered LBW in newborn babies, which included both preterm and intrauterine growth retardation. In addition, estimates from a backward stepwise method with a probability of a retention cutoff of 20% (aRR: 1.85 (1.14–3.07); adjusting for term pregnancy, maternal education, mode of delivery, BMI category and antenatal visits) and 5% remained within the variability range (aRR: 1.72 (1.04–2.85); adjusting for term pregnancy and mode of delivery) (full analysis is not presented) across five models. Thus, overfitting might not have any impact on the true association. Despite all these limitations, the study had several strengths, such as a high-quality serological assessment in the international accredited laboratory, as well as an accurate measurement of birth weight and pregnant maternal height and weight, and a robust and systematic use of statistical methods, including the estimation of relative risk.

## 4. Materials and Methods

### 4.1. Study Design, Study Site and Population

The study was conducted between July 2014 and December 2015 in the rural Mirzapur subdistrict, Tangail, Bangladesh, where the living population were routinely followed by an ongoing demographic surveillance system. The study site is supported by a 750 bedded rural tertiary hospital, named Kumudini Women’s Medical College and Hospital, with a unique and separate inpatient obstetric care and outpatient maternity clinic. Women aged 11–49 years who have a recent history (within the past 21 days) of spontaneous or habitual abortion or stillbirth or intrauterine death, constituted as cases. The study was of a longitudinal design where 208 age-matched (±2.5 years) pregnant women of the same gestation (±2 weeks) were followed until delivery to record the pregnancy outcome, as well as to measure the newborn’s weight.

### 4.2. Ethical Approval

The study received full, unconditional approval from the Research Review Committee and the Ethical Review Committee of icddr,b (protocol number: PR-14037). Informed written consent was taken explaining the intentions of the study from each of the study-enrolled participants before the formal interview, collection of blood samples and anthropometric assessments. We obtained written, informed consent from caregivers/parents of the participating children before enrollment.

### 4.3. Anthropometric Assessment and Collection of Blood Sample

All pregnant women were evaluated for the measurement of their weight and height by trained study personnel using TANITA, HD-314 for the weight (nearest to 100 g) and locally made standardized wooden height scales with 0.1 cm precision. Their body mass index [BMI = {weight (kg)/height (m)^2^}] was calculated accordingly. Five ml of blood was collected in a red stoppered vacutainer by venipuncture from all the study participants at enrollment who gave written consent by the trained laboratory technician. Serum was separated and stored at 2–8 °C immediately, or stored at −20 °C for longer preservation. All specimens were transported to the Molecular and Serodiagnostic Laboratory of International Centre for Diarrhoeal Disease Research, Bangladesh (icddr,b), Dhaka by maintaining a cold temperature (4–8 °C) and standard laboratory procedure for serological assessment.

### 4.4. Assessment and Definition of Serological Profiles for Toxoplasmosis

All serological profiles were assessed using chemiluminescent immunoassay, which was conducted in the Immulite-1000 (SIEMENS, Seattle, WA, USA), an automated immunochemistry analyzer, to assess the levels of *T. gondii* IgG and IgM, scrupulously adhering to the manufacturer’s instructions. A cutoff of 6.5 IU/mL for IgG and 0.9 IU/mL for IgM were considered as positive for *T. gondii* infection. A positive *T. gondii* IgG indicated a past or current infection. On the other hand, *T. gondii* IgM positive results indicated a recent infection (primary/acute, reactivation or reinfection) and an IgM negative result indicated that the pregnant women are yet to experience a recent infection. Serological profiles for other pathogens of the TORCH (rubella, cytomegalovirus, herpes simplex-1 and 2) panel were also assessed using the same method.

### 4.5. Major Outcome and Co-Variates

Newborn babies with LBW were treated as the main outcome variable. LBW is defined as a newborn baby with a birth weight less than or equal to 2499 g, according to the criteria of the World Health Organization [47]. This study considered LBW irrespective of gestational age or intrauterine growth retardation. Several maternal sociodemographic, pregnancy morbidities and other characteristics were considered to further contribute to understanding the confounding and modifying effects between a major exposure and outcome. Among these included: pregnant women aged less than 20 years, the education level of the mothers, household wealth quintiles (equated following principal component analysis methods [48]), number of living children with the mother, the mode of delivery, maternal anemia, the BMI category of the pregnant mother (normal, undernourished or overweight/obese), the duration of pregnancy at enrolment and term birth.

### 4.6. Statistical Analysis

The distribution, crude and adjusted associations between *T. gondii* infection and other co-variates and LBW (outcome variable) were determined using a modified Poisson regression [49] with robust variance for the estimation of relative risks and their 95% confidence intervals. For our analysis, newborns with LBW were the outcome variable while seroprevalence of anti- *T.gondii* IgM or IgG was the independent variable.

A series of multiple regression analyses were considered to assess the confounding and modifying effects of these variables, according to the following statistical models, namely: Model-1: *T. gondii* infection; Model-2: Model-1 with maternal, age, education, family size and wealth index; Model-3: Model-2 with an antenatal visit, maternal anemia and mode of delivery; Model-4: Mode-3 with BMI category; Model-5: Model-4 with a length of gestation at enrolment and term pregnancy.

A number of sensitivity analyses were performed to understand the accuracy of the observed association from a different model, such as the assessment of potential confounding and modifying effects. No confounding effect was observed, although the educational level of the pregnant women may be a potential modifier. Thus, to assess the modifying effect, an interaction term (educational level of the pregnant women × *T. gondii*) was used with Model-5 (Table S1). The clinical importance of the *T. gondii* and the overall discriminating capacity of the models was assessed by estimating the area under the receiver operating characteristics curve (AROC) (unadjusted and adjusted). The AROC represented plotting the fraction of true positives (i.e., sensitivity) versus the fraction of false positives (i.e., one-specificity), corresponding to each value of a model indicated how well the predicted score discriminates the risk of LBW between pregnant women with or without *T. gondii* infection. For example, an AROC of 0.5 indicated that screening of *T. gondii* infection to predict the risk of LBW had no ability to discriminate; whilst an AROC of 0.70 indicated that 70% of the time, a pregnant woman drawn at random was at risk of delivering an LBW baby if she is exposed to *T. gondii* with other possible risk factors. For each model, overall sensitivity, specificity, positive and negative predictive values were estimated to predict LBW babies delivered by the pregnant women with and without *T. gondii* infection. Due to the lack of an equation method, the discriminating capacity for LBW was estimated by performing logistic regression (Supplementary Table S4). For further assessment of potential confounders and modifiers, a backward stepwise approach was also explored with the level of exclusion at 20% and 5% to assess the variation of estimated risk determined by the various models. Finally, to maintain the consistency with the existing literature [6,8], an association between *T. gondii* infection and LBW was further assessed by excluding IgM+; IgG+ and IgM+; IgG− (Table S2).

In addition, a number of the goodness-of-fit tests were performed, including Pseudo R-squired, which accounted for the amount of variance explained by each model, such as Akaike’s information criteria (AIC), Bayesian information criteria (BIC), Hosmer–Lemeshow goodness (*p*-value) (for logistic regression) and Pearson’s goodness-of-fit (*p*-value) (for modified Poisson regression). All data analyses were performed using STATA (Version 14, Stata Corp, College Station, TX, USA).

## 5. Conclusions

There was a strong association between *T. gondii* infection among rural Bangladeshi women with LBW of newborns. Such a strong significant association requires further investigation following strong research methodology in both high and low *T. gondii* seroprevalence sites with a rigorous assessment of all possible confounders and modifiers to establish the causal relationship. Strong evidence is yet to be established on the potential benefits of routine assessment for *T. gondii* infection during pregnancy. Moreover, further studies are also important to investigate the path of vertical transmission in terms of adverse or fatal risk of *T. gondii* infection to formulate appropriate preventive strategies. Furthermore, future studies should be designed in an area with higher LBW among newborns with proper environmental and behavioral variables to understand why LBW in newly born babies is not reducing in Bangladesh, expectedly for decades.

## Figures and Tables

**Table 1:** Distribution of *T. gondii* immunoglobulins (IgM and IgG) in women by birthweight of their newborns.

	Normal Birth Weight (n = 169)		Low Birth Weight (n = 39)	
	N	%	N	%
IgM−; IgG−	132	78.1	24	61.5
IgM−; IgG+	30	17.8	15	38.5
IgG+; IgM+	2	1.2	0	0.0
IgM+; IgG−	5	3.0	0	0.0

**Table 2:** Distribution and crude associations of maternal factors by newborn birth weight.

	Normal Birth Weight (n = 169)	Low Birth Weight (n = 39)	Unadjusted Relative Risk (95% CI)
Age group			
<20 years	18.34	17.95	1.00 (0.43–2.35)
20–29 years	55.03	53.85	Ref.
≥30 years	26.63	28.21	1.07 (0.51–2.21)
Pregnant women’s education			
>9 years of schooling	32.54	20.51	Ref.
5–9 years of schooling	60.36	58.97	1.45 (0.65–3.24)
<5 years of schooling	7.1	20.51	3.15 (1.18–8.39)
Wealth quintiles			
Poor	24.85	12.82	Ref.
Lower middle	17.16	20.51	2.03 (0.66–6.21)
Middle	15.98	28.21	2.72 (0.95–7.83)
Upper middle	18.93	20.51	1.88 (0.62–5.75)
Rich	23.08	17.95	1.43 (0.45–4.51)
Number of under 18 years children			
No child	35.5	23.08	Ref.
1–2 children	37.87	41.03	1.53 (0.68–3.47)
≥3 children	26.63	35.9	1.82 (0.79–4.2)
Antenatal visit			
No	60.36	64.1	0.88 (0.46–1.69)
Yes	39.64	35.9	Ref.
Reported anemia			
No	5.92	2.56	Ref.
Yes	94.08	97.44	2.12 (0.29–15.45)
Mode of delivery			
Normal Vaginal Delivery	47.34	71.79	Ref.
Caesarian section	52.66	28.21	0.42 (0.21–0.85)
Pregnant women’s education BMI			
Normal	64.5	53.85	Ref.
Under nutrition	8.28	15.38	1.86 (0.75–4.60)
Over nutrition/obese	27.22	30.77	1.28 (0.63–2.60)
Gestation at enrollment (weeks) *	19.84 ± 10.08	17.84 ± 8.99	0.98 (0.95–1.01)
Term pregnancy (>36 weeks)			
No	80.47	43.59	Ref.
Yes	19.53	56.41	3.60 (2.07–6.27)

* mean ± standard deviation (duration of pregnancy at enrolment); CI: Confidence interval; Ref.: reference.

**Table 3:** Association ^1^ between *T. gondii infection* and low birth weight.

	Model 1	Model 2	Model 3	Model 4	Model 5
*T. gondii*					
IgM−; IgG−	Ref.	Ref.	Ref.	Ref.	Ref.
IgG+ or IgM+	1.88 (1.07–3.3)	1.76 (1.01–3.07)	1.76 (1.02–3.02)	1.78 (1.03–3.06)	2.00 (1.17–3.42)
Age group					
<20 years		1.04 (0.5–2.16)	1.57 (0.65–3.82)	1.54 (0.59–4.00)	1.48 (0.43–5.11)
20–30 years		Ref.	Ref.	Ref.	Ref.
≥30 years		0.95 (0.5–1.8)	0.71 (0.3–1.7)	0.70 (0.32–1.54)	0.50 (0.24–1.05)
Pregnant women’s education					
>9 years of schooling		Ref.	Ref.	Ref.	Ref.
5–9 years of schooling		1.5 0 (0.75–3.02)	1.29 (0.69–2.44)	1.34 (0.70–2.56)	1.02 (0.54–1.93)
<5 years of schooling		3.52 (1.52–8.17)	2.7 (1.14–6.36)	3.10 (1.24–7.77)	2.35 (1.05–5.27)
Wealth quintiles					
Poor		Ref.	Ref.	Ref.	Ref.
Lower middle		1.83 (0.63–5.3)	1.77 (0.6–5.21)	1.72 (0.60–4.92)	1.02 (0.36–2.91)
Middle		3.05 (1.16–8.02)	3.27 (1.2–8.91)	3.31 (1.19–9.22)	3.15 (1.18–8.41)
Upper middle		2.18 (0.76–6.28)	2.41 (0.84–6.89)	2.71 (0.89–8.28)	1.36 (0.45–4.07)
Rich		1.87 (0.61–5.78)	2.18 (0.68–7.01)	2.43 (0.72–8.14)	2.04 (0.63–6.61)
Number of under 18 years children					
No children			Ref.	Ref.	Ref.
1–2 children			1.96 (0.8–4.8)	2.00 (0.77–5.18)	1.78 (0.56–5.63)
≥3 children			2.3 (0.78–6.81)	2.28 (0.76–6.79)	2.58 (0.74–9.00)
Antenatal visit			Ref.		
No			0.76 (0.42–1.39)	0.72 (0.38–1.35)	0.59 (0.34–1.05)
Yes				Ref.	Ref.
Reported anemia					
No			Ref.	Ref.	Ref.
Yes			1.76 (0.31–9.97)	1.57 (0.26–9.57)	1.40 (0.19–10.27)
Mode of delivery					
Normal Vaginal Delivery			Ref.	Ref.	Ref.
Caesarian section			0.48 (0.25–0.93)	0.46 (0.24–0.9)	0.29 (0.14–0.59)
Pregnant women’s BMI					
Normal				Ref.	Ref.
Under nutrition				1.54 (0.59–4.00)	1.68 (0.87–3.26)
Over nutrition/obese				0.70 (0.32–1.54)	1.96 (0.94–4.09)
Gestation at enrollment (weeks)					0.99 (0.96–1.02)
Term pregnancy (>36 weeks)					
No					4.66 (2.51–8.68)
Yes					Ref.
Model fitness					
Pseudo R-squared	0.016	0.062	0.097	0.109	0.195
AIC	209.142	215.731	218.265	219.661	205.758
BIC	215.817	249.106	268.328	276.401	269.171
Pearson goodness-of-fit (*p*-value)	0.972	0.931	0.941	0.874	0.948

^1^ Relative risk (95% Confidence interval); Ref.: reference; Pseudo R^2^ = squired accounted for the amount of variance explained by each model. AIC: Akaike’s information criteria; BIC: Bayesian information criteria. Outcome: Low birth weight. Model-1: *T. gondii* infection; Model-2: Model-1 with women’s age, education, family size and wealth index; Model-3: antenatal visit, maternal reported anemia, mode of delivery; Model-4: Model-3 with BMI category; Model-5: Model-4 with gestation at enrolment and term pregnancy.

## Data Availability

The dataset contained personal information of the study participants. Our institutional review board will not disclose this kind of information. Thus, our policy is not to make available the dataset in the manuscript, the supplemental files, or a public repository. However, part of dataset related to this manuscript is available upon request and readers may contact Armana Ahmed (aahmed@icddrb.org (accessed on 25 January 2022) from the Research Administration & Strategy of icddr,b to request the data (http://www.icddrb.org/ (accessed on 25 January 2022).

## Supplementary Materials

The following supporting information can be downloaded at: https://www.mdpi.com/article/10.3390/pathogens11030336/s1, Table S1: Association 1 between T. gondii infection and low birth weight (effect modification due to pregnant women’s education); Table S3: Distribution of other viruses of the TORCH panel and their association with low birth weight; Table S4: Corresponding association ^1^ between *T. gondii* infection and low birth weight from logistic regression model (Estimates for AROC).

## Abbreviations

AIC	Akaike’s information criteria
AROC	Area under the receiver operating characteristics curve
BMI	Body mass index
BIC	Bayesian information criteria
CI	Confidence interval
Icddr,b	International Centre for Diarrheal Disease Research, Bangladesh
Ig	Immunoglobulin
LBW	Low birth weight
OR	Odds ratio
RR	Relative risk
