# Supplementary material for: Toxoplasma gondii Infection Is Associated with Low Birth Weight: Findings from an Observational Study among Rural Bangladeshi Women
Source: Pathogens. 2022 Mar 10;11(3):336. doi: 10.3390/pathogens11030336 (PMC8954211; doi:10.3390/pathogens11030336)
Supplement: Supplementary file 1 [file pathogens-11-00336-s001.zip › pathogens-1603406-supplementary.pdf]

pathogens

MDPI

Supplementary Materials

Table S1. Association [1] between *T. gondii* infection and low birth weight (effect modification due to pregnant women's education).

T. gondii	
IgM–; IgG–	Ref.
IgG+/IgM+	4.88 (1.74–13.69)
Age group	
<20 years	1.23 (0.36–4.19)
20–30 years	Ref.
≥30 years	0.63 (0.28–1.42)
Pregnant women's education	
>9 years of schooling	Ref.
5–9 years of schooling	1.79 (0.74–4.32)
<5 years of schooling	5.12 (1.86–14.06)
Wealth quintiles	
Poor	Ref.
Lower middle	1.25 (0.45–3.52)
Middle	2.96 (1.11–7.9)
Upper middle	1.38 (0.46–4.12)
Rich	2.04 (0.64–6.51)
Number of under 18 years children	
No children	Ref.
1–2 children	1.49 (0.47–4.72)
≥3 children	2.11 (0.6–7.38)
Antenatal visit	
No	0.58 (0.33–1.02)
Yes	Ref.
Reported anaemia	
No	Ref.
Yes	1.73 (0.27–11.17)
Model of delivery	
Normal Vaginal Delivery	Ref.
Caesarian section	0.32 (0.16–0.64)
Pregnant women's BMI	
Normal	Ref.
Under nutrition	1.62 (0.86–3.05)
Over nutrition/obese	1.75 (0.85–3.6)
Gestation at enrollment (weeks)	0.99 (0.96–1.02)
Term pregnancy (>36 weeks)	
No	4.25 (2.26–7.99)
Yes	Ref.
Interaction (Toxoplasma x Mothers education)	0.38 (0.14–0.98)
Pseudo R^2	0.208

[1] Relative risk (95% Confidence interval); Ref.: reference; Pseudo R^2-squired accounted for the amount of variance explained by each model.

**(N = 203)**	**RR (95% CI)**
T. gondii	

IgM–; IgG–	Ref.
IgG+; IgM–	2.46 (1.42–4.25)
Age group	
<20 years	1.75 (0.55–5.59)
20–30 years	Ref.
≥30 years	0.45 (0.21–0.97)
Mothers education	
>9 years of schooling	Ref.
5–9 years of schooling	1.06 (0.56–2)
<5 years of schooling	2.26 (1–5.12)
Wealth quintiles	
Poor	Ref.
Lower middle	0.95 (0.34–2.68)
Middle	3.16 (1.2–8.37)
Upper middle	1.22 (0.41–3.62)
Rich	1.78 (0.55–5.81)
Number of under 18 years children	
No children	Ref.
1–2 children	1.95 (0.66–5.78)
≥3 children	2.81 (0.86–9.15)
Antenatal visit	
No	0.55 (0.31–0.96)
Yes	Ref.
Maternal reported anaemia	
No	Ref.
Yes	1.5 (0.2–11.23)
Model of delivery	
Normal Vaginal Delivery	Ref.
Caesarian section	0.32 (0.16–0.65)
Mothers BMI	
Normal	Ref.
Under nutrition	1.56 (0.82–2.98)
Over nutrition/obese	2.22 (1.05–4.66)
Gestation at enrollment (weeks)	0.99 (0.96–1.02)
Term pregnancy (>36 weeks)	
No	4.98 (2.7–9.19)
Yes	Ref.
Pseudo R^2	0.206
AIC	201.41
BIC	264.18
Pearson goodness-of-fit (*p* value)	0.95

[1] Relative risk (95% Confidence interval); Ref.: reference.

Table S3. Distribution of other viruses of the TORCH panel and their association with low birth weight.

	Normal Birth Weight (n = 169)		Low Birth Weight (n = 39)		RR (95% CI)
	**N**	**%**	**N**	**%**	
Rubella					
IgM–; IgG–	35	20.7	4	10.3	Ref.
IgM–; IgG+	131	77.5	35	89.7	2.06 (0.73–5.78)

IgG+; IgM+	3	1.8	0	0.0	-
IgM+; IgG–	0	-	0	-	
Cytomegalovirus					
IgM–; IgG–	0	-	0	-	-
IgM–; IgG+	148	87.6	34	87.2	-
IgG+; IgM+	21	12.4	5	12.8	1.03 (0.40–2.63)
IgM+; IgG–	0	-	0	-	-
Herpes simplex-1					
IgM–; IgG–	4	2.4	2	5.1	Ref.
IgM–; IgG+	53	31.4	14	35.9	0.63 (0.14–2.76)
IgG+; IgM+	94	55.6	18	46.2	0.48 (0.11–2.08)
IgM+; IgG–	18	10.7	5	12.8	0.65 (0.13–3.36)
Herpes simplex-2					
IgM–; IgG–	101	59.8	25	64.1	Ref.
IgM–; IgG+	40	23.7	9	23.1	0.93 (0.43–1.98)
IgG+; IgM+	4	2.4	0	0.0	-
IgM+; IgG–	24	14.2	5	12.8	0.87 (0.33–2.27)

Table S4. Corresponding association [1] between *T. gondii* infection and low birth weight from logistic regression model (Estimates for AROC).

	**Model_1**	**Model_2**	**Model_3**	**Model_4**	**Model_5**
Odds ratio	2.23 (1.06–4.68)	2.19 (1.00–4.79)	2.14 (0.94–4.83)	2.15 (0.94–4.91)	2.93 (1.14–7.55)
Pseudo R-square	0.02	0.08	0.13	0.15	0.27
Hosmer–Lemeshow goodness (*p* value)		0.17	0.80	0.41	0.03
AIC	200.43	203.85	204.62	205.12	183.62
BIC	207.10	237.22	254.68	261.85	247.03

[1] Odds ratio (95% Confidence interval); Pseudo R-squired accounted for the amount of variance explained by each model. Outcome: Low birth weight. Mode- 1: *T. gondii* infection. Model-2: Model-1 with women age, education, family size and wealth index. Model-3: Model-2 with antenatal visit, maternal reported anemia, mode of delivery. Model-4: Model-3 with BMI category. Model-5: Model-4 with gestation at enrolment and term pregnancy. AIC: Akaike's information criteria; BIC: Bayesian information criteria.
